# Amaurosis Caused by Crowding of Teeth

**Published:** 1869-08

**Authors:** 


Amaurosis Caused by Crowding of Teeth.—Mr. Hancock (*Lancet*) reports the following peculiar case; a boy, aged eleven, whose sight had been previously unimpaired, found upon waking one morning that his sight was entirely lost. He was admitted to the Charing-Cross Hospital about a month afterwards, when it was found that his teeth were much crowded and wedged together; the jaws, in fact, not being large enough for them. Two permanent and four milk molar teeth were extracted, and the boy could distinguish light from darkness on the same evening; on the following morning he could make out objects. Eleven days after he was discharged cured, the only treatment beyond the removal of the teeth being two doses of aperient medicine.

